# Postprandial Hypertriglyceridemia Is Associated with the Variant 54 Threonine *FABP2* Gene

**DOI:** 10.3390/jcdd5030047

**Published:** 2018-09-13

**Authors:** María Fatima Garcés Da Silva, Yamil Adrian Guarin, Yenny Carrero, Hilda Stekman, María Luisa Núñez Bello, Celsy Hernández, Rafael Apitz, Mercedes Fernández-Mestre, Germán Camejo

**Affiliations:** 1 Associated Research Laboratorio de Investigaciones Básicas y Aplicadas, Departamento de Bioquímica, Escuela de Bioanálisis, Facultad de Medicina, Universidad Central de Venezuela, Caracas 48321, Venezuela; adrian_guarin@hotmail.com (Y.A.G.); yenny_gab@hotmail.com (Y.C.); hstekman@yahoo.com (H.S.); mluisanunezb@gmail.com (M.L.N.B.); celsyhernandez@gmail.com (C.H.); 2 National Academy of Medicine, Caracas 41421, Venezuela; rapitz@gmail.com; 3 Laboratorio de Fisiopatología, Centro de Medicina Experimental, Instituto Venezolano de Investigaciones Científicas, Caracas 21827, Venezuela; mfernandezmestre@gmail.com; 4 Associated Research Clinical Chemistry Laboratory, Department Laboratory Medicine, Karolinska Institute, 14186 Stockholm, Sweden; german.camejo@telia.com

**Keywords:** FABP2 gene polymorphism, oral fat tolerance test, normolipidemic non-obese healthy subjects, hypertriglyceridemia

## Abstract

**Purpose:** Fasting or postprandial hypertriglyceridemia is considered an independent cardiovascular disease (CVD) risk factor. The intestinal fatty acid binding protein (FABP2) is involved in the intracellular transport and metabolism of fatty acids. The presence of the *Ala54Thr* polymorphism of the *FABP2* gene appears to be involved in postprandial hypertriglyceridemia. We explored the possible association of the Ala54Thr polymorphism with fat intolerance in apparently healthy, fasting, normolipidemic subjects with normal body-mass index and without diabetes. **Methodology:** A total of 158 apparently healthy individuals were classified as fat tolerant (*n* = 123) or intolerant (*n* = 35) according to their response (plasma triglycerides) to an oral abbreviated tolerance test with blood samples taken at 0, 2 and 4 h. At 0 h, all subjects ingested 26.3 g of fats. Presence of the Ala54Thr polymorphism of the *FABP2* gene was evaluated by polymerase chain reaction–restriction fragment length (PCR–RFLP). **Results:** The group with fat intolerance (postprandial hypertriglyceridemia group) showed an increased frequency of the Thr54Thr genotype when compared with the group with normal fat tolerance (control group) (23% vs. 4%, respectively, OR: 16.53, 95% CI: 4.09–66.82, *p*: 0.0001, pc: 0.0003). Carriers of at least one Thr54 allele were up to six times more prevalent in the fat intolerant group than in the non-carriers. (OR: 6.35; 95% CI: 1.86–21.59, *p*: 0.0003, pc: 0.0009). The levels of plasma triglycerides (Tg) at 4 h after the test meal were higher in carriers of at least one 54Thr allele than in carriers of the Ala54 allele (p < 0.05). **Conclusions:** There is a significant association between postprandial hypertriglyceridemia and the presence of at least one 54Thr allele of the *FABP2* gene. In addition, subjects with this genotype showed an increased ratio of Tg/HDL-cholesterol. This parameter is a marker of increased CVD risk and insulin resistance.

## 1. Introduction

High postprandial plasma triglycerides are associated with increased risk of atherosclerotic cardiovascular disease (CVD) and all cause death [1,2,3,4,5]. Furthermore, epidemiological studies show that increased non-fasting triglyceride (Tg) levels are an independent risk factor for CVDs [6,7]. However, only fasting Tg concentration is routinely measured in the clinical setting. Thus, ignoring the potential role of postprandial hypertriglyceridemia in the evaluation of CVD risk. We found recently that a simplified oral fat tolerance test (OFTT) measuring plasma Tg at 0, 2 and 4 h after a test meal can detect fat intolerance in a cohort of apparently healthy, non-obese subjects with normal fasting lipids [8]. The fat intolerant subjects showed increased ratios of apoB/apoAI, Tg/HDL-C, Total cholesterol/HDL-C and levels of remnant cholesterol. All these parameters are thought to be associated with an increased risk of CVD [8].

The plasma levels and characteristics of postprandial lipoproteins can be modulated during their synthesis and secretion by the enterocytes. The intestinal fatty acids binding protein IFABP, or FABP2, is a key protein for the transport of absorbed fatty acids to cytoplasm sites where Tg and phospholipids are synthesized and assembled into lipoprotein particles [9]. A single nucleotide polymorphism (SNP) in codon 54 of the *FABP2* gene leads to the amino acid substitution of alanine (Ala54: wild variant) for threonine (Thr54: mutated variant). The Thr54 variant protein has a higher binding affinity for fatty acids than the normal variant. In addition, the carriers of this variant show increased frequency of fasting hypertriglyceridemia that appears associated with CVD risk and type 2 diabetes [9,10,11,12,13]. However, not all studies confirmed these associations, probably because the biochemical effects of the Ala54Thr polymorphism are modulated by other genes with dissimilar ethnic distribution [14,15,16,17].

In the present report we explored the possible association of the Ala54Thr polymorphism of the *FABP2* gene with the results of a simplified fat tolerance test that can detect associations of markers of CVD risk even in healthy, non-obese, subjects with normal fasting lipids. Our results indicate that the Ala54Thr polymorphism is significantly more prevalent in subjects with postprandial fat intolerance that also show lipoprotein markers of increased CVD risk [8].

## 2. Methods

### 2.1. Studied Subjects’ Cohort

The studied sample included one hundred and fifty-eight (158) apparently healthy individuals (96 women and 62 men), with normal weight and with ages between 20 and 59 years, who agreed to participate in the study. The subjects were residents of Great Caracas and attended the Laboratory of Basic & Applied Research at the Bioanalysis School of the Central University of Venezuela. DNA samples for the study were obtained from biologically unrelated individuals of admixed urban population [18]. Both parents and the grandparents of the subjects studied were born in Venezuela.

Individuals with fasting plasma triglycerides above 150 mg/dL or with plasma cholesterol above 250 mg/dL, hypertension or hypertension treatment, or smokers (self-reported), chronic alcohol consumption (self-reported) were excluded. Furthermore, patients with established diagnosis of cardiomyopathies, diabetes and inflammatory diseases or with their treatment in the previous six months were excluded.

### 2.2. Bioethical Norms

All the participants signed a written consent for the study in which the possible risks and the potential benefits of their participation was explained. The study protocol was carried out under the ethical standards established by WHO for research on humans and the Helsinki declaration ratified by the 29th World Medical Assembly, Tokyo, 1995. The protocol was approved by the Ethics Committee of the University Hospital Caracas (HUC).

### 2.3. Sample Collection

A fasting (~14 h) sample of 10 mL of blood was drawn by venipuncture, after antisepsis of the anterior arm and taking the steps and considerations necessary to prevent venous stasis and hemolysis. The samples were collected in a tube with ethylenediaminetetraacetic acid (EDTA) as anticoagulant and another tube without anticoagulant. Then, a breakfast with a total load of 26.3 g of fat present in a cheese patty and coffee was given. Samples of 10 mL of blood were taken after 2 and 4 h after ingestion of the breakfast. The blood samples were centrifuged (600× *g* for 15 min) in a refrigerated centrifuge at 4 °C within a period not exceeding 30 min. Serum and plasma were stored in aliquots at −70 °C until assayed.

### 2.4. Biochemical Parameters

Glucose, cholesterol and triglycerides were determined with automated equipment (Modular Analytics, Roche Diagnostics). Cholesterol in lipoproteins was analyzed with automated electrophoretic equipment (Helena Laboratories SAS-1). Insulin was assayed using an adsorbent enzyme immunoassay (ELISA), (DRG International, Marburg, Germany). Apolipoproteins (apo) AI and apoB were evaluated by a radial immunodiffusion gel-based method (LTA RID, s.r.l., Bussero, Italy).

### 2.5. FABP2 Genotype Analysis

**Figure 1:**
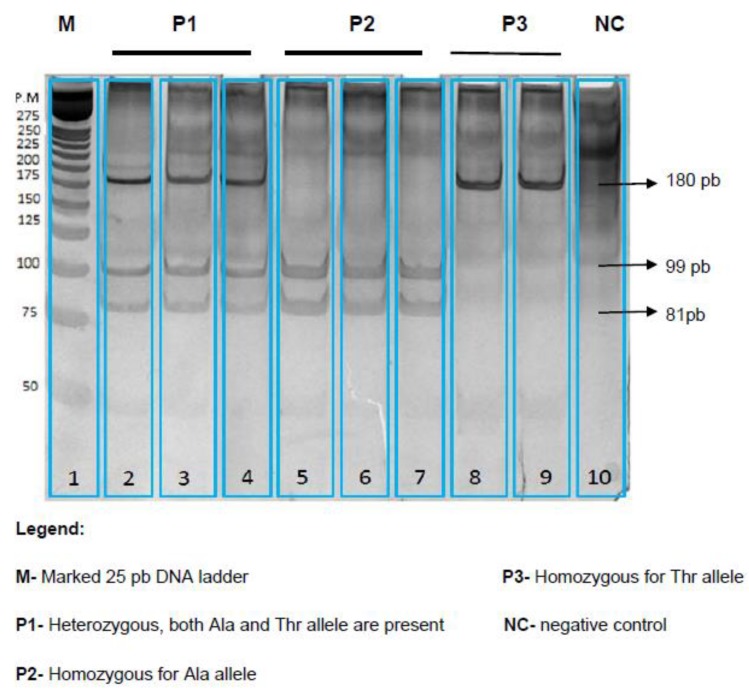
Genotyping of the *FABP2* (rs1799883) Ala54Thr polymorphism. After digestion of the polymerase chain reaction PCR product with *Hha*I in a polyacrylamide-gel electrophoresis stained with silver nitrate, P1 samples heterozygotes have three bands, at 180 bp, 99 bp and 81pb, P2 homozygotes have two bands at 99 bp and 81 bp. P3 homozygotes have one band at 180 bp.

DNA extraction and purification was performed by the method of Bunce modified [19]. The polymorphic variant Ala54Thr (rs1799883) of the *FABP2* gene was studied by PCR-RFLP technique, using primers and restriction enzymes as reported by Ribalta et al. [20]. For the genotyping, the sequence of the forward and reverse primers used was 5′-ACAGGTGGTAATATAGTGAAAAG-3′ and 5′-TACCCTGAGTTCAGTTCCGTC-3′. The PCR conditions were as follows: Initial denaturation at 95 °C for 5 min was followed by 35 cycles of PCR under the following conditions: denaturation at 95 °C for 1 min, annealing at 52 °C for 1 min, and extension at 72 °C for 1 min. A final extension step at 72 °C for 10 min followed the last PCR cycle. The PCR product (180 bp) was digested with restriction enzyme *Hha*I (Invitrogen, Carlsbad, CA, USA). The digested samples were separated by gel electrophoresis and visualized by polyacrylamide-gel electrophoresis stained with silver nitrate; the gel was visualized in a digital photo-documentation system, Uvitec equipment (Gel Documentation Uvitec Limited, Bio-Rad, Hercules, CA, USA) as depicted in Figure 1. PCR products having an intact *Hha*I site are cleaved into 99 and 81 bp fragments, whereas the Ala54Thr substitution abolishes the restriction site.

For quality control, and to confirm the restriction digestions, direct sequencing was performed to screen these polymorphisms in 5–10% of randomly selected samples. The results showed 100% concordance, suggesting no error in restriction digestion methods (Forensic and Genetic Studies Unit of IVIC). The assignment of genotypes was done using control samples with known genotypes (as positive controls) and a control without enzyme (as negative control) after each digestion step.

### 2.6. Statistical Analysis

Biochemical data were evaluated using SPSS v17 (IBM) program and were analyzed following the recommendations of the International Federation of Clinical Chemistry [21]. Descriptive statistical analysis of the continuous variables, and the test for evaluation of distribution and equality of variances were analyzed with the PRISM v.6 package (GraphPad^®^ Software). Most of the studied biochemical variables complied with a near-normal distribution (D’Agostino and Pearson test) and showed no significant differences in the variances of the 2 groups compared. The similar variances are reflected in the similar standard deviations (SDs). The close values for the SD suggest that the subjects of the studied sample came from a population with continuous variables with a near-Gaussian distribution. To compare the means of variables studied with the same test, the results are reported as mean plus or minus SDs, and all the differences were analyzed with the 2-tailed, unpaired Mann–Whitney non-parametric test. The variables in the fat-tolerant and fat-intolerant groups were considered significantly different when *p* levels were <0.05. Notice that we are evaluating differences between these 2 groups.

Allelic and genotypic frequencies were determined by direct counting. The chi-square test was used with MAXLIK program to evaluate the Hardy–Weinberg (H–W) equilibrium model. The statistical significance of allele frequency differences between groups was estimated chi-square test using 2 × 2 contingency tables. The Bonferroni corrected *p*-values (pc) were obtained by multiplying the *p*-values by the total number of variables analyzed and were considered significant when *p* < 0.05 [22]. Relative risk with corresponding 95% confidence intervals (95% CI) was calculated as odds ratios (OR) according to tool for the analysis of association studies [23].

The difference between the averages of biochemical variables for the genotypes was determined by student *t*-test. Partial correlation coefficients were calculated to examine the linear relationships between biochemical variables and the influence of the genotypes on postprandial triglyceride responses and it was evaluated by the area under the curve (AUC) at 0, 2 and 4 h after the test breakfast, using the trapezoidal rule. A *p*-value < 0.05 was considered statistically significant.

## 3. Results

### 3.1. General Characteristics of Individuals Studied

The study involved 158 unrelated healthy subjects (39% men, 61% women; with a mean age of 31.0 ± 11.1 years, with normal fasting cholesterol and Tg classified, according to their postprandial Tg concentrations, in fat tolerant individuals (control group, *n* = 123) and fat intolerant individuals (*n* = 35). An individual was considered intolerant to fat when the increase in the concentrations of Tg at 2 h and 4 h post-load was greater than 30% of baseline [8]. For all biochemical variables, the normality assumption was fulfilled.

In Table 1, the arithmetic mean is shown as the value of central tendency and the standard deviation (SD) estimated with a confidence interval of 95%. When comparing the average total glucose, insulin and cholesterol between the two groups no significant difference (*p* > 0.05) was observed. However, average LDL-C, VLDL-C and Apo B were significantly increased in the intolerance fat group compared to the control group (*p* < 0.05). In contrast, the average HDL-C was significantly increased in the control group and decreased in the intolerance fat group (*p* < 0.05). With respect to basal and postprandial Tg at 2 h and 4 h and Tg AUC, it was observed that the mean values were significantly higher in the group with fat intolerance than in the group with normal fat tolerance (*p* < 0.001).

### 3.2. Frequency of FABP2 Polymorphisms in Normal Tolerance and Intolerance Fat Group

Table 2 shows the frequencies of *FABP2* genotypes and alleles in the total group and in the fat tolerant and fat intolerant groups. The results indicate that there was Hardy–Weinberg equilibrium for the genotype distribution of Ala54Thr (*p* < 0.05) in the total group, as well as in the groups classified according to postprandial Tg concentrations into fat tolerant or fat intolerant. There was a significantly increased frequency of the Thr54Thr genotype in the fat intolerant group when compared with the fat tolerant group (control group) (23% vs. 4%, respectively, OR: 16.53, 95% CI: 4.09–66.82, *p*: 0.0001, pc: 0.0003). In contrast, a significantly increased frequency of the Ala54Ala genotype was observed in the control group compared to the group with postprandial hypertriglyceridemia (50% vs. 17%, respectively). In the fat intolerant group there was an increased frequency of subjects that carried at least one Thr54 allele (Ala54Thr + Thr54Thr) than in the fat tolerant group (Ala54Ala), (83% vs. 50%, respectively, OR: 6.35; 95% CI: 1.86 to 21.59, *p*: 0.0003, pc: 0.0009).

### 3.3. Correlation of FABP2 Polymorphism and Biochemical Parameters and BMI

**Figure 2:**
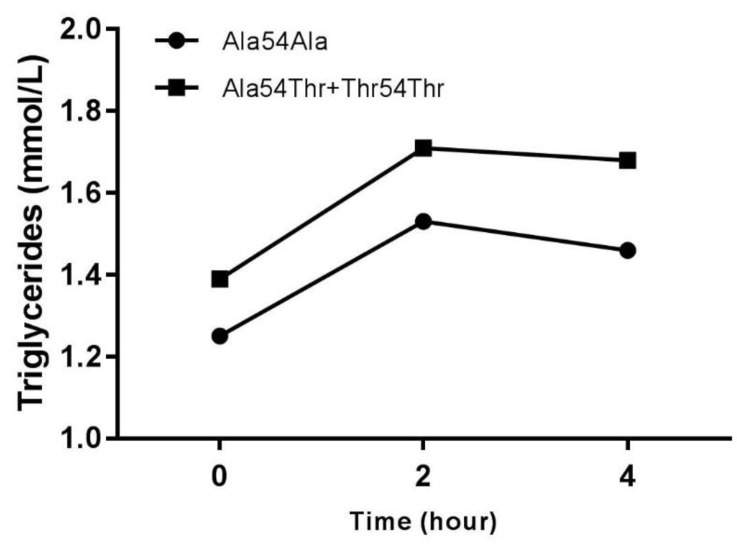
Effect of *FABP2* Ala54Thr polymorphism on triglycerides (mmol/L) after OFTT.

Table 3 shows the biochemical parameters in the total individuals grouped according to genotypes. As can be observed, the triglyceride’s concentrations in fasting as well as triglycerides postprandial 2 h and 4 h, triglycerides (AUC), the ratio triglycerides/HDL-C and BMI are significantly increased in individuals that carried at least one Thr54 allele (Ala54Thr + Thr54Thr genotypes) compared to the Ala54Ala genotype. Figure 2 shows the curves for plasma triglycerides at 0, 2 and 4 h after the test meal for the subjects classified according to genotypes. Plasma Tg at 0, 2 and 4 h were higher in individuals that carried at least one Thr54 allele (Ala54Thr + Thr54Thr).

The results of the partial correlation coefficients analysis for the genotypes carriers of at least one Thr54 allele (Ala54Thr + Thr54Thr) revealed a significant positive correlation with fasting triglycerides, triglycerides postprandial 4 h, triglycerides (AUC), the ratio triglycerides/HDL-C and BMI. In contrast, the Ala54Ala genotype was significantly negative correlated with fasting triglycerides, triglycerides postprandial 4 h, triglycerides (AUC) and the ratio triglycerides/HDL-C (Table 4).

## 4. Discussion

The *FABP2* gene has been proposed as a candidate gene for the development of diabetes and insulin resistance because the encoded protein is involved in the absorption and metabolism of fatty acids [9,24]. In addition, the Ala54Thr polymorphism of *FABP2* is also associated with fasting hypertriglyceridemia and obesity. There are controversial results about the association of the Ala54Thr polymorphism with the postprandial response to dietary fats. Ågren et al. found in subjects with high BMI and high plasma insulin an increased Tg response to a fat oral test [25]. On the other hand, Pratley et al. showed an augmented modest response of plasma fatty acids to a fat oral test but not of plasma Tg in Pima Indians with the Thr54 polymorphism [26]. In subjects with type 2 diabetes Georgopoulus et al. [11] showed that the postprandial Tg response in a fat tolerance test was linearly correlated with the dose of the Thr54 allele. Our study examined the association of this polymorphism with postprandial Tg in a cohort of apparently healthy, young (31 ± 11 years) subjects with normal fasting lipids, normal plasma insulin and normal BMI. Our results show a significant association between the presence of the Ala54Thr *FABP2* gene polymorphism and postprandial hypertriglyceridemia. We believe that our results are also the first to show that a simplified fat intolerance test in subjects with normal fasting lipids can identify the relation of the Ala54Thr genotype with lipoprotein markers of CVD risk. In addition, we observed that individuals with at least one Thr54 allele (genotype Thr54Thr or Ala54Thr) have higher frequency of postprandial hypertriglyceridemia. We observed that in these subjects the TG/HDL-C ratio, is also significantly correlated with the Thr54 genotype. This ratio is associated with an increasingly atherogenic lipid phenotype [27].

The mechanisms by which the Ala54Thr polymorphism in *FABP2* increases the frequency of a prolonged Tg response are not established. It is not clear how the sequence changes that augment its affinity for fatty acids can increase the intestinal secretion of Tg-rich lipoprotein particles and/or decrease its intravascular lipolysis [9]. Perhaps other proteins participate in the postprandial Tg response. It will be important to evaluate in our cohort the associations with apoCIII secretion and the various isoforms of apoE [9].

This is the first study evaluating polymorphisms of the *FABP2* gene in a Venezuelan apparently healthy population of mixed ethnicity. Compared to apparently healthy subjects, from Europe, Asia and African-Americans, and aborigines from Chile, with our Venezuelan cohort the Ala54Thr polymorphism has a higher prevalence in the sample tested (48%), however, the Thr54Thr frequencies are similar (8%) [28,29,30,31].

The present study has several limitations. The main one is the relatively small number of subjects studied. In addition, the possible link of the Ala54Thr polymorphism with effects in lipoproteins that may be associated with long term lipoprotein markers of CVD risk need to be further explored in studies with a prospective design, in order to ascertain if such association translates into atherosclerotic cardiovascular disease. Finally, the apparent ethnic differences in the polymorphism distribution may limit the general validity of our conclusions.

## 5. Conclusions

Hypertriglyceridemia can be caused by genetic and/or environmental factors. Our results suggest that the Ala54Thr polymorphism (rs1799883) of the *FABP2* gene may be one of the genetic factors that contribute to the development of postprandial hypertriglyceridemia. In the studied population sample this seems to be associated with increased lipoprotein markers of CVD risk.

## Figures and Tables

**Table 1:** Anthropometric and biochemical parameters for all the studied subjects and after their classification into fat tolerant and fat intolerant group.

Biochemical Parameters	All Subjects	Fat Tolerances	Fat Intolerances
BMI (kg/m^2^)	22.8 ± 2.4	22.4 ± 2.4	23.6 ± 2.7 *
Insulin (pmol/L)	61.8 ± 27.8	59.7±26.6	65.2 ± 39.1
Fasting glucose mmol/L)	4.84 ± 0.44	4.81 ± 0.45	4.89 ± 0.42
Total Cholesterol (mmol/L)	4.78 ± 0.98	4.76 ± 1.03	4.97 ± 0.82
HDL-C (mmol/L)	1.3 ± 0.44	1.35 ± 0.45	1.06 ± 0.37 **
VLDL-C (mmol/L)	0.52 ± 0.17	0.5 ± 0.17	0.61 ± 0.18 **
LDL-C (mmol/L)	2.91 ± 0.82	2.86 ±0.84	3.19 ± 0.77 *
Fasting Tg (mmol/L)	1.33 ± 0.41	1.24 ± 0.38	1.63 ± 0.38 ***
Tg postprandial 2 h (mmol/L)	1.63 ± 0.55	1.43± 0.40	2.34 ± 0.41 ***
Tg postprandial 4 h (mmol/L)	1.58 ± 0.60	1.37 ± 0.40	2.34 ± 0.42 ***
Triglycerides AUC (mmol * h/L)	6.18 ± 1.99	5.48 ± 1.46	8.66 ± 1.59 ***
Remnants Cholesterol (mmol/L)	0.59 ± 0.21	0.56 ± 0.21	0.73 ± 0.20 **
Apo A1 (g/L)	1.15 ± 0.23	1.17 ± 0.23	1.09 ± 0.21
Apo B (g/L)	1.03 ± 0.24	0.99 ± 0.25	1.17 ± 0.36 *
Ratio Tg/HDL-C	2.31 ± 1.25	2.00 ± 0.96	3.39 ± 1.53 ***
Ratio Total Cholesterol/HDL	4.07 ± 1.49	3.76 ± 1.14	5.16 ± 2.01 **
Ratio Apo B/ApoA1	0.89 ± 1.04	0.84 ± 1.09	1.07 ± 1.71 **

“Data from Garcés et al.” [8]. Abbreviations: HDL-C, high-density lipoprotein cholesterol; LDL-C, low-density lipoprotein cholesterol; VLDL-C, very-low-density lipoprotein cholesterol; Tg, triglycerides; AUC, area under the curve. Data are expressed as means ± SD. Only tolerant and intolerant were statistically evaluated with the student *t*-test with significance level α = 0.05. * *p* < 0.05 ** *p* < 0.005 *** *p* < 0.0001.

**Table 2:** *FABP2* genotype and alleles frequencies in individuals with fat tolerances and fat intolerants.

FABP2 Ala54Thr Polymorphism Genotype	Normal Tolerance Fats Group *n* = 123	Intolerance Fats Group *n* = 35	OR (IC 95%)	pc
**Codominant inheritance model**				
Ala54Ala	50.4 (62)	17.1 (6)	1.00 (Reference)	
Ala54Thr	45.5 (56)	60.0 (21)	3.87 (1.46–10.29)	0.0003 *
Thr54Thr	4.1 (5)	22.9 (8)	16.53 (4.09–66.82)	
**Dominant inheritance model**				
Ala54Ala Ala54Thr + Thr54Thr	50.4 (62) 49.6 (61)	17.1 (6) 82.9 (29)	1.00 (Reference) 6.35 (1.86–21.59)	0.0009 *
**Recessive inheritance model**				
Ala54Ala + Ala54Thr	95.9 (118)	77.1 (27)	1.00 (Reference)	0.0039 *
Thr54Thr	4.1 (5)	22.9 (8)	6.99 (2.12–23.06)	
Alleles Ala54	73.2 (180)	47.1 (33)	1.00 (Reference)	0.00013 *
Thr54	26.8 (66)	52.9 (37)	3.05 (1.76–5.28)	

Note. The genotype and alleles frequencies are expressed in percentages, followed by the number of individuals or chromosomes in parentheses. OR: Odds ratio, pc: Corrected *p*-values, ns: Not significant, *: Significant.

**Table 3:** Biochemical parameters according to *FABP2* genotype.

Biochemical Variables	Genotype		
	Ala54Ala	Ala54Thr + Thr54Thr	Statistical Significance
BMI (kg/m^2^)	22.2 ± 2.4	23.0 ± 2.5	*p* < 0.05
Fasting glucose (mmol/L)	4.85 ± 0.38	4.91 ± 0.49	n.s.
Insulin (pmol/L)	62.4 ± 27.2	60.8 ± 28.3	n.s.
Total Cholesterol (mmol/L)	4.75 ± 1.04	4.80 ± 0.94	n.s.
Fasting Tg (mmol/L)	1.25 ± 0.38	1.39 ± 0.42	*p* < 0.05
Tg postprandial 2 h (mmol/L)	1.53 ± 0.48	1.71 ± 0.59	*p* < 0.05
Tg postprandial 4 h (mmol/L)	1.46 ± 0.48	1.68 ± 0.68	*p* < 0.05
Triglycerides AUC (mmol * h/L)	5.77 ± 1.70	6.50 ± 2.13	*p* < 0.05
HDL-C (mmol/L)	1.33 ± 0.48	1.25 ± 0.40	n.s.
LDL-C (mmol/L)	2.84 ± 0.86	2.97 ± 0.79	n.s.
VLDL-C (mmol/L)	0.51 ± 0.16	0.54 ± 0.19	n.s.
Ratio Tg/HDL-C	2.12 ± 1.20	2.45 ± 1.28	*p* < 0.05
Apo B (g/L)	1.02 ± 0.26	1.04 ± 0.31	n.s.

Abbreviations: BMI, Body mass index; Tg, triglycerides; AUC, area under the curve; HDL-C, high-density lipoprotein cholesterol; LDL-C, low-density lipoprotein cholesterol; VLDL-C, very-low-density lipoprotein cholesterol; Apo B, Apolipoprotein B; Significant values (*p* < 0.05); n.s, non-significant.

**Table 4:** Partial correlation coefficients between *FABP2* genotype biochemical parameters and body mass index (BMI).

Biochemical Variables	Genotype		
	Ala54Ala	Ala54Thr + Thr54Thr	Statistical Significance
BMI (kg/m^2^)	−0.177	0.177	*p* < 0.05
Fasting glucose (mmol/L)	−0.076	0.076	n.s.
Insulin (pmol/L)	0.025	−0.025	n.s.
Total Cholesterol (mmol/L)	−0.014	0.014	n.s.
Fasting Tg (mmol/L)	−0.196	0.196	*p* < 0.01
Tg postprandial 4 h (mmol/L)	−0.24	0.24	*p* < 0.005
Triglycerides AUC (mmol * h/L)	−0.236	0.236	*p* < 0.005
HDL-C (mmol/L)	0.126	−0.126	n.s.
LDL-C (mmol/L)	−0.087	0.087	n.s.
VLDL-C (mmol/L)	−0.073	0.073	n.s.
Ratio Tg/HDL-C	−0.204	0.204	*p* < 0.01
Ratio TC/HDL-C	−0.132	0.132	n.s.
Apo B (g/L)	−0.011	0.011	n.s.

Abbreviations: BMI, Body mass index, Tg, triglycerides; TC, Total Cholesterol; AUC, area under the curve; HDL-C, high-density lipoprotein cholesterol; LDL-C, low-density lipoprotein cholesterol; VLDL-C, very-low-density lipoprotein cholesterol. Partial correlation coefficients controlling for age and sex. Significant values (*p* < 0.05); n.s, non-significant compared to Ala54Ala genotype.
